# Effects of pulse parameters on the temperature distribution of a human head exposed to the electromagnetic pulse

**DOI:** 10.1038/s41598-021-02396-8

**Published:** 2021-11-25

**Authors:** Shan Wang, Zhongguo Song, Yanning Yuan, Guozhen Guo, Jianjun Kang

**Affiliations:** 1 Faculty of Automation and Information Engineering, Xi’an University of Technology, Xi’an, 710048 China; 2 Department of Radiation Biology, Air Force Medical University, Xi’an, 710032 China; 3 Xi’an Jiushuo Institute of Biotechnology, Xi’an, 710065 China

**Keywords:** Computational biophysics, Biophysics, Biological physics

## Abstract

The presence of blood–brain barrier (BBB) is a major obstacle to effectively deliver therapeutics to the central nervous system (CNS); hence, the outcomes following treatment of CNS diseases remain unsatisfactory. Fortunately, electromagnetic pulses (EMPs) provide a non-invasive method to locally open the BBB. To obtain the optimal pulse parameters of EMP-induced BBB opening to ensure the effective delivery of CNS drugs, it is particularly important to measure and assess the effects of pulse parameters on the temperature distribution in the human head exposed to EMPs. In this paper, the specific anthropomorphic mannequin phantom was adopted and the temperature increase in the human head induced by EMPs of different parameters was estimated in the software “COMSOL Multiphysics”. The results show that the temperature distribution profiles with different EMP parameters have almost similar characteristics, the highest temperature increase values in the human head are positively correlated with variations of EMP parameters, and potential hazards to the human head may occur when EMP parameters exceed the safety threshold, which will provide theoretical basis for seeking the optimal EMP parameters to open the BBB to the greatest extent within a safe range.

## Introduction

**Figure 1:**
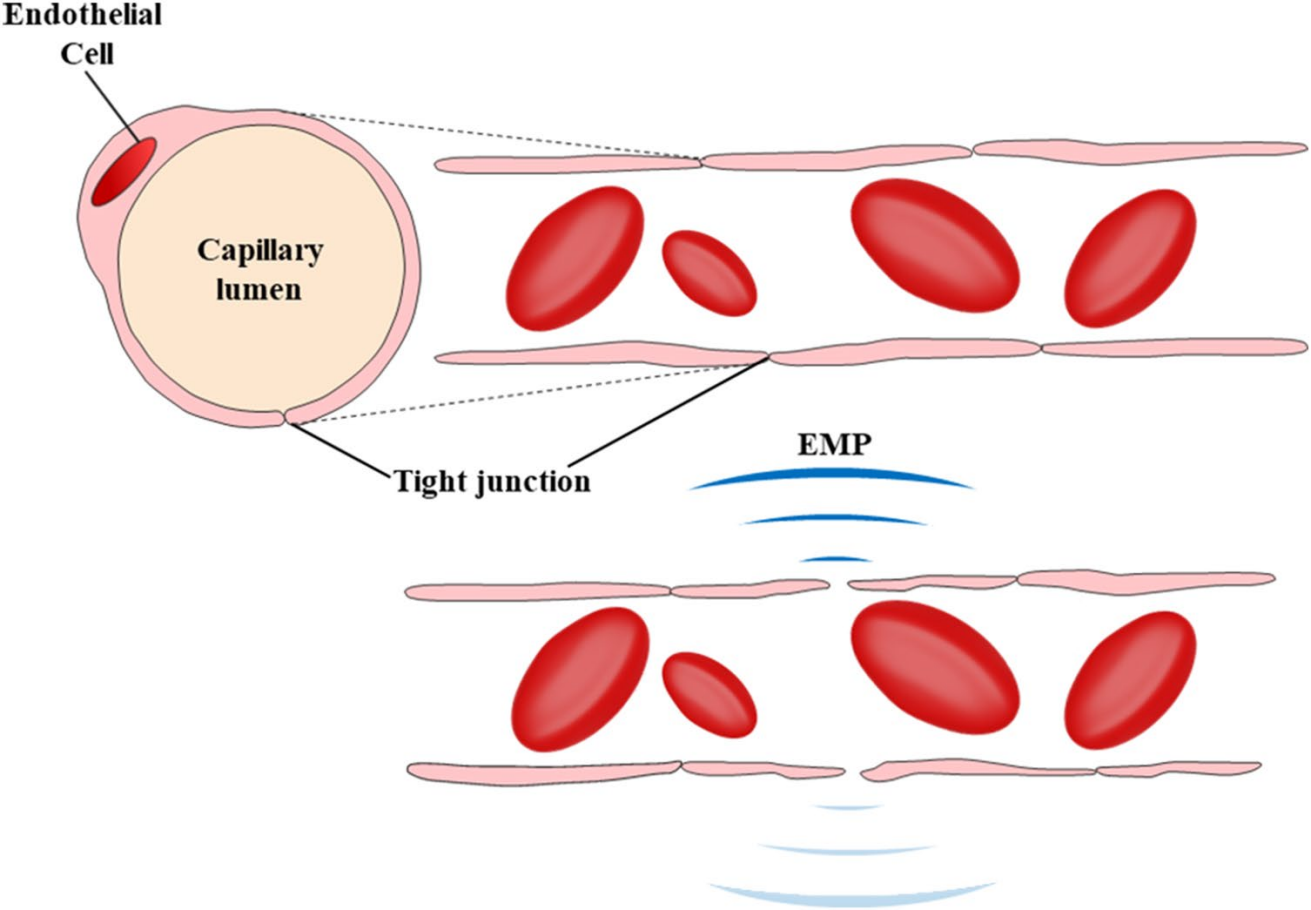
The schematic of BBB opening following EMPs. Without EMP (top) and with EMP (bottom). We used 3D Studio Max software, version 2020 (https://www.autodesk.com/products/3ds-max/overview) for the image.

With the aggravation of ambient air pollution and the ageing of the population, central nervous system (CNS) diseases, such as Alzheimer’s disease (AD), Parkinson’s disease (PD), stroke, and neurodevelopmental disorders have become a serious and common public health concern^1–3^. Meanwhile, an increasing incidence of CNS diseases has been reported from numerous studies^4–9^. Considerable work has been done to develop therapeutics for these diseases. However, the number of potential therapeutics that can reach the criteria of success is abysmally low due to the complexity of the CNS drug development, and one of the biggest challenges in the development of therapeutics for CNS disorders is achieving sufficient blood–brain barrier (BBB) penetration^10–13^. Previous studies have shown that an electromagnetic pulse (EMP) could open the BBB locally, transiently and reversibly^14–16^, and the schematic of BBB opening caused by EMP is shown in Fig. 1. The use of EMP-facilitated drug delivery to the brain by transiently increasing the permeability of the BBB is somewhat risky. Like any medical intervention, adverse effects are always the priority to deal with. Although EMP-induced BBB opening appears promising, EMP parameters must be properly controlled to avoid side effects.

As is known to all, passing through a biological system, electromagnetic radiation (EMR) can be reflected, transmitted, refracted or absorbed, and it has an important influence on lossy-dielectric materials such as biological tissues in which result thermal energy increase^17^. Absorption of EMR energy stirs up the motion of charged particles and rotation of molecules, primarily water, and is converted into heat, inevitably causing a rise in tissue temperature^18^. According to the amount of energy absorbed by the biological system, effects of EMR on the biological system can be divided into thermal and non-thermal. Thermal effects occur with the temperature increase exceeding 1 °C causing cellular and intracellular changes particularly at molecular level^17^. Sufficient electromagnetic fields (EMF) exposure will trigger a thermal effect that can heat the human body, leading to DNA damage, mutations and cell death. Even if the heat emitted does not cause a thermal effect and is not enough to heat the human body, it may still cause great damage to cells, and this damage can also be converted into diseases, such as cancer. In the RF /MW risk assessment, according to ANSI (1981), interactions inducing a temperature increase lower than 0.5 °C in the human body are commonly accepted, even by the WHO. And WHO, IEEE and ICNIRP assure that we can be protected against all health effects due to RF/MWs under such a threshold^19^. Therefore, besides the pronounced increase in BBB permeability, the temperature increase in biological tissues should also be evaluated when performing the study of EMP-induced BBB opening and it should be ensured that EMP will not cause irreversible adverse effects on the organism when it opens the BBB to the maximum extent. Prevalent researches on EMP-induced BBB opening remain committed to explore the optimal pulse parameters to enhance the BBB permeability greatly. Regrettably, recent articles only focused on the experimental studies (rats, etc.), and demonstrated a variety of parameters that affect the BBB opening extent^14–16^. However, the temperature increases in the tissues induced by EMPs and the resulting adverse effects have rarely been taken seriously, and the research in this field is almost blank.

This article breaks through the limitations of existing experimental research (animals only), investigates the temperature increase in the human head exposed to EMPs of different parameters due to the probable negative effects of EMR on the human health, and obtains the safety threshold of EMPs on the human head, which would provide theoretical basis for seeking the optimal EMP parameters to make the BBB disruption to the greatest extent within a safe range.

## Models and methods

### The model and simulation scene

The human head geometry is the same as that of the specific anthropomorphic mannequin (SAM) phantom provided by IEEE, IEC, and CENELEC from their standard specification of SAR value measurements^20^, and it is widely used in simulations for RF wave propagation. Table 1 provides the important parameters that are used for SAM head model in simulations.

**Table 1:** The using parameters of the simulation.

Parameters	Value
Permittivity for the brain tissue	58.13
Conductivity for the brain tissue	1.15 S/m
Density of the brain tissue	1030 kg/m^3^
Heat capacity of blood	3639 J/(kg*K)
Density of blood	1000 kg/m^3^
Frequency	250 MHz

**Figure 2:**
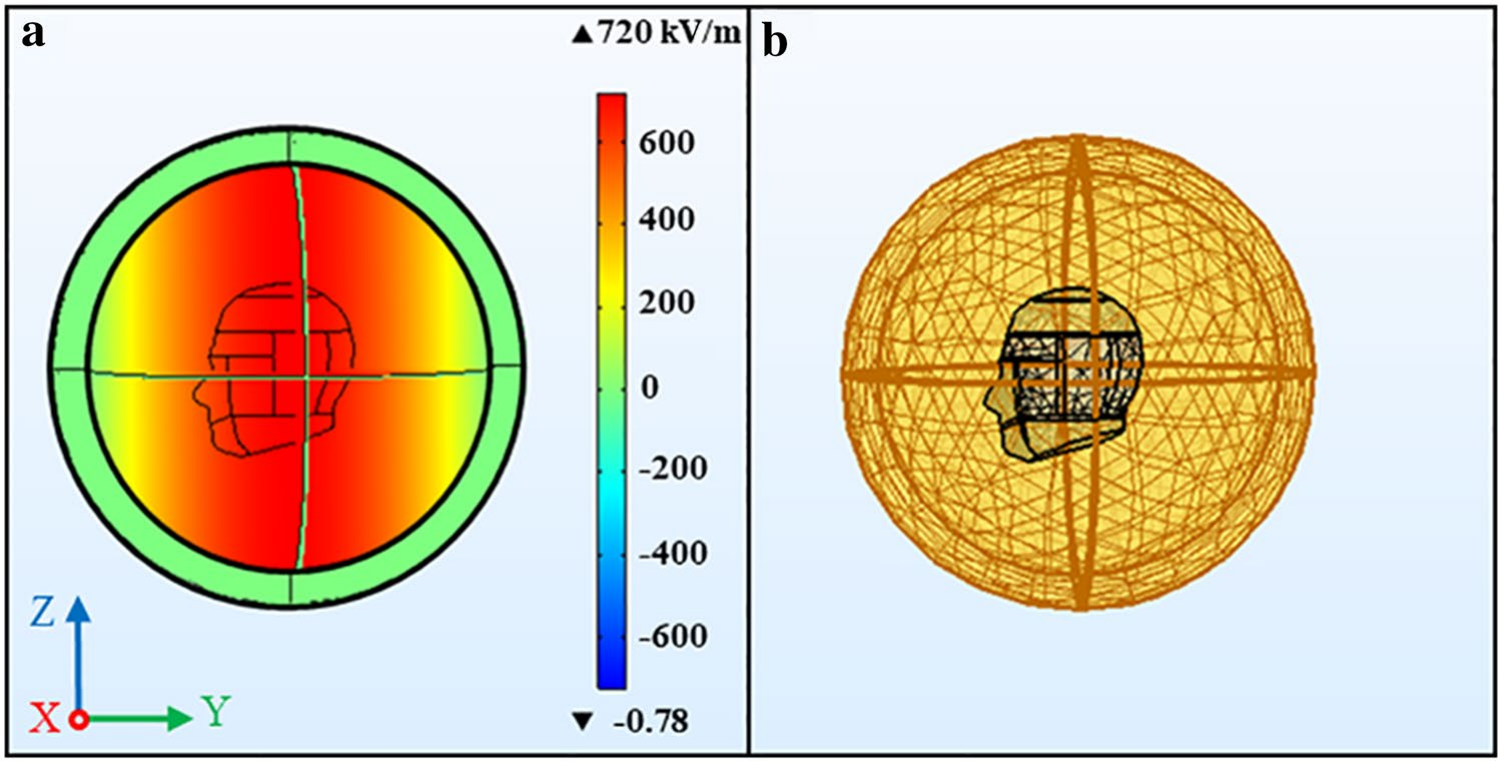
The simulation scene and model meshing. (**a**) Coordinate system definition and the incident wave, (**b**) simulation region and FEM meshing. We used COMSOL Multiphysics software, version 5.4 (https://www.comsol.com/comsol-multiphysics) for simulation results.

The simulation scene and model meshing are shown in Fig. 2. As depicted in Fig. 2a, the background plane wave propagates in the negative direction of the y axis (the posterior to anterior of the head), and the electric field is polarized along the z axis. The incident field is periodic Gaussian pulses with field intensity 720 kV/m, the pulse total number 800, pulse width 40 ns, and the pulse repeat frequency 10 Hz, which denoted by E = 720 kV/m, n = 800, τ = 40 ns and f = 10 Hz respectively. The boundary adopts the scattering boundary condition (SBC), and the spherical surface domains furthest from the center of the sphere are the perfectly matched layers (PMLs). All domains other than the human head are defined as air. Figure 2b exhibits the simulation region and finite element method (FEM) meshing of the SAM model, and simulations are performed on this FEM meshing model.

### Theoretical methodology

COMSOL Multiphysics is a powerful, interactive environment. For the propagation of electromagnetic fields, the software uses the proven FEM and performs finite element analysis, adaptive meshing, and error control by using a variety of numerical solvers; therefore, it is suitable for evaluating human head exposure to an EMP.

In this research, COMSOL Multiphysics software, version 5.4 (https://www.comsol.com/comsol-multiphysics) was used for modelling of the temperature distribution and simulation of the temperature increase in the human head induced by EMP radiation. The step-by-step solution was performed. Firstly, the frequency domain solver was used to calculate the electric field distribution and electromagnetic loss in the head, and the numerical simulation was carried out at 250 MHz. Then, the transient solver was used to estimate temperature rise in the head, and where, the generalized heat source and convective heat flux were adopted.

The propagation of the electromagnetic wave in the human head satisfies the Maxwell’s equation^21,22^, which mathematically describes the interdependence of the electromagnetic waves. The electromagnetic field penetrated in the human head is demonstrated as the following equation:

1 $$\nabla \times \mu_{r}^{ - 1} (\nabla \times {\mathbf{E}}) - k_{0}^{2} \left( {\varepsilon_{r} - \frac{j\sigma }{{\omega \varepsilon_{0} }}} \right){\mathbf{E}} = {\mathbf{0}}$$

where ${\mathbf{E}}$ is the electric field intensity (V/m), $\mu_{r}$ is the relative magnetic permeability of the material, $\varepsilon_{r}$ is the relative dielectric constant, $\sigma$ is the material conductivity, and $k_{0}$ is the free space wave number (m^−1^).

The absorbed energy is converted into thermal energy and causes the rise in temperature of the biological tissues. For the purpose of heat transfer analysis, the temperature distribution within the human head is obtained using the Penne’s bioheat equation^21–23^ as follows.

2 $$\rho C_{\rho } \frac{\partial dT}{{\partial t}}{ + }\rho C_{\rho } {\mathbf{u}} \cdot \nabla dT{ + }\nabla \cdot ( - k\nabla dT) = Q{ + }\rho_{b} C_{\rho ,b} \omega_{b} (T_{b} - dT) + Q_{met}$$

where $\rho$ is the tissue density (kg/m^3^), $C_{\rho }$ is the heat capacity of tissues (J/kg/K), $k$ is the thermal conductivity of tissues (W/m/K), $dT_{{}}$ is the tissue temperature (℃), $T_{b}$ is the temperature of the blood (℃), $\rho_{b}$ is the density of the blood (kg/m^3^), $C_{\rho ,b}$ is the heat capacity of the blood (3960 J/kg/K), $\omega_{b}$ is the blood perfusion rate (1/s), $Q_{met}$ is the metabolism heat source (W/m^3^), and $Q$ is the external heat source (electromagnetic heat-source density) (W/m^3^).

Taking into account the uncertainty of EMP parameters and the impact of these variations on simulation results, the simulation program allows changing the basic settings and monitoring the temperature distribution in the head. In order to obtain the temperature increase in the artificial head when varying EMP parameters, the process of the human head exposure to EMPs was established and simulated, and the electric field and temperature distribution in the human head were calculated.

## Results

Due to the characteristics of EMPs such as periodicity, short duration, large number of pulses and long overall action time, COMSOL simulations were used to reflect the cumulative effect of multi-period electromagnetic pulse signals, so as to study the effect of electromagnetic pulses on the human head temperature. It can effectively analyze not only the dose–effect relationship between some pulse physical parameters (the pulse field intensity, the pulse width) and the temperature increase, but also the time-related factor (the pulse number) and the temperature increase. In this part of the study, the temperature increases with various EMP parameters have been simulated by FEM based on computer software.

### The temperature distribution and the specific absorption rate with benchmark parameters

**Figure 3:**
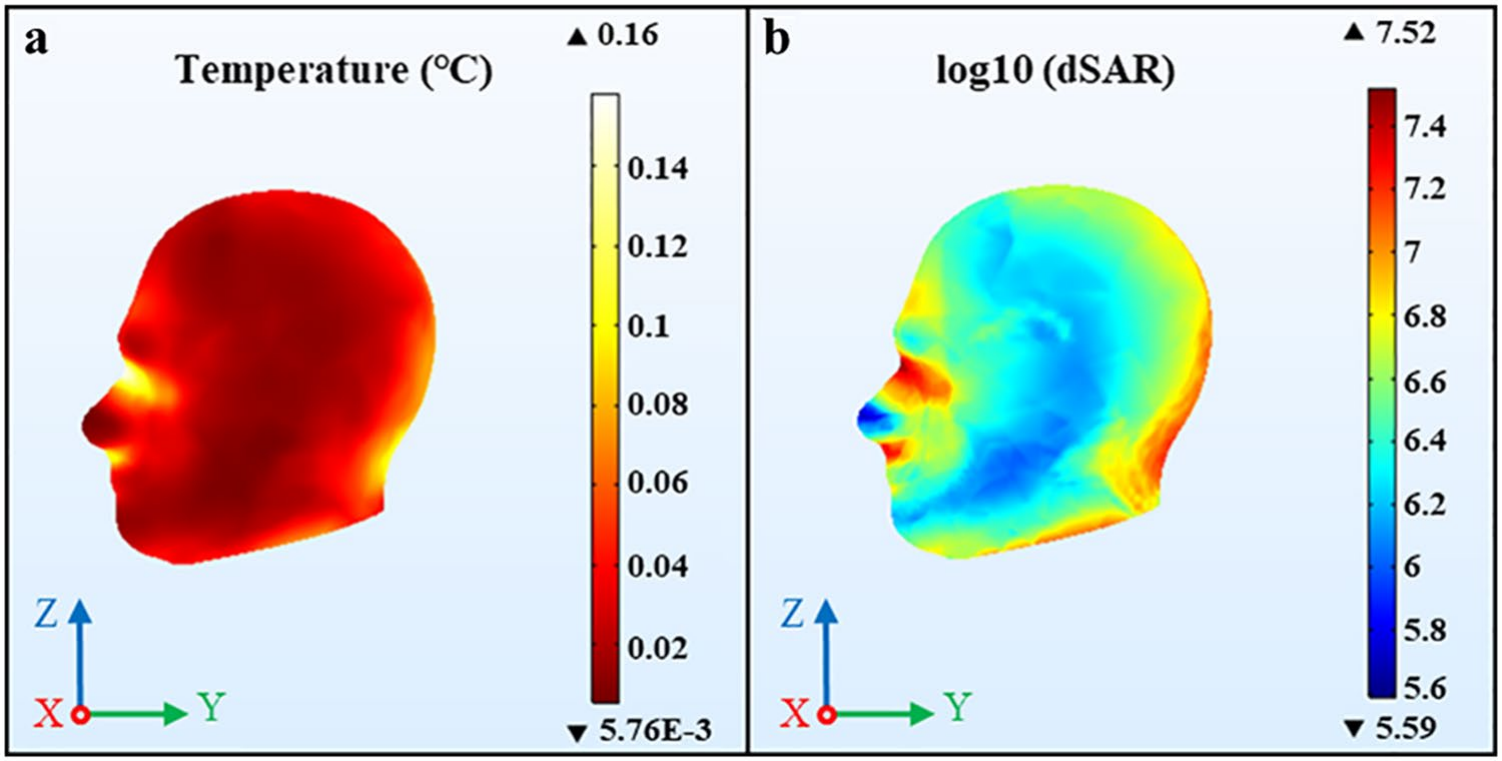
The temperature distribution and SAR values distribution of the head with benchmark parameters. (**a**) The temperature distribution, (**b**) the SAR distribution. We used COMSOL Multiphysics software, version 5.4 (https://www.comsol.com/comsol-multiphysics) for simulation results.

The temperature distribution and the specific absorption rate (SAR) distribution of the head exposed to an EMP of 250 MHz are shown in Fig. 3 according to the parameters used by other sub-group experiments (E = 720 kV/m, n = 800, τ = 40 ns, f = 10 Hz), which are used as the benchmark parameters in this study.

As it is clearly seen from Fig. 3 that with the benchmark parameters, the highest temperature increase value in the human head is measured as 0.16 °C (non-thermal effect). The surface of the human head closer to the incident source exerts obvious temperature increase, and it significantly decreases as the human head deepens.

### The electric field norm and the electromagnetic loss

The multi-section background electric field is shown in Supplementary Video. The three-dimension multi-section electric field in the human head after an EMP exposure with the frequency 250 MHz is shown in Supplementary Fig. S1. Supplementary Fig. S1a shows the multi-section electric field norm in the head and Supplementary Fig. S1b displays the electromagnetic loss.

As obtained from Supplementary Fig. S1a, the transmitted field is the highest at the surface of the SAM head and it decreases promptly with increasing distance into the model when exposed to an EMP of 250 MHz, and the peak electric field strength at a certain point in space is nearly 6.6 times stronger compared with the incident field. It can be seen from Supplementary Fig. S1b that when exposed to an EMP, the electromagnetic power loss density is approximately 3.39E10 W/m^3^.

### The dose–effect between the temperature increase of the human head and the pulse field intensity

**Figure 4:**
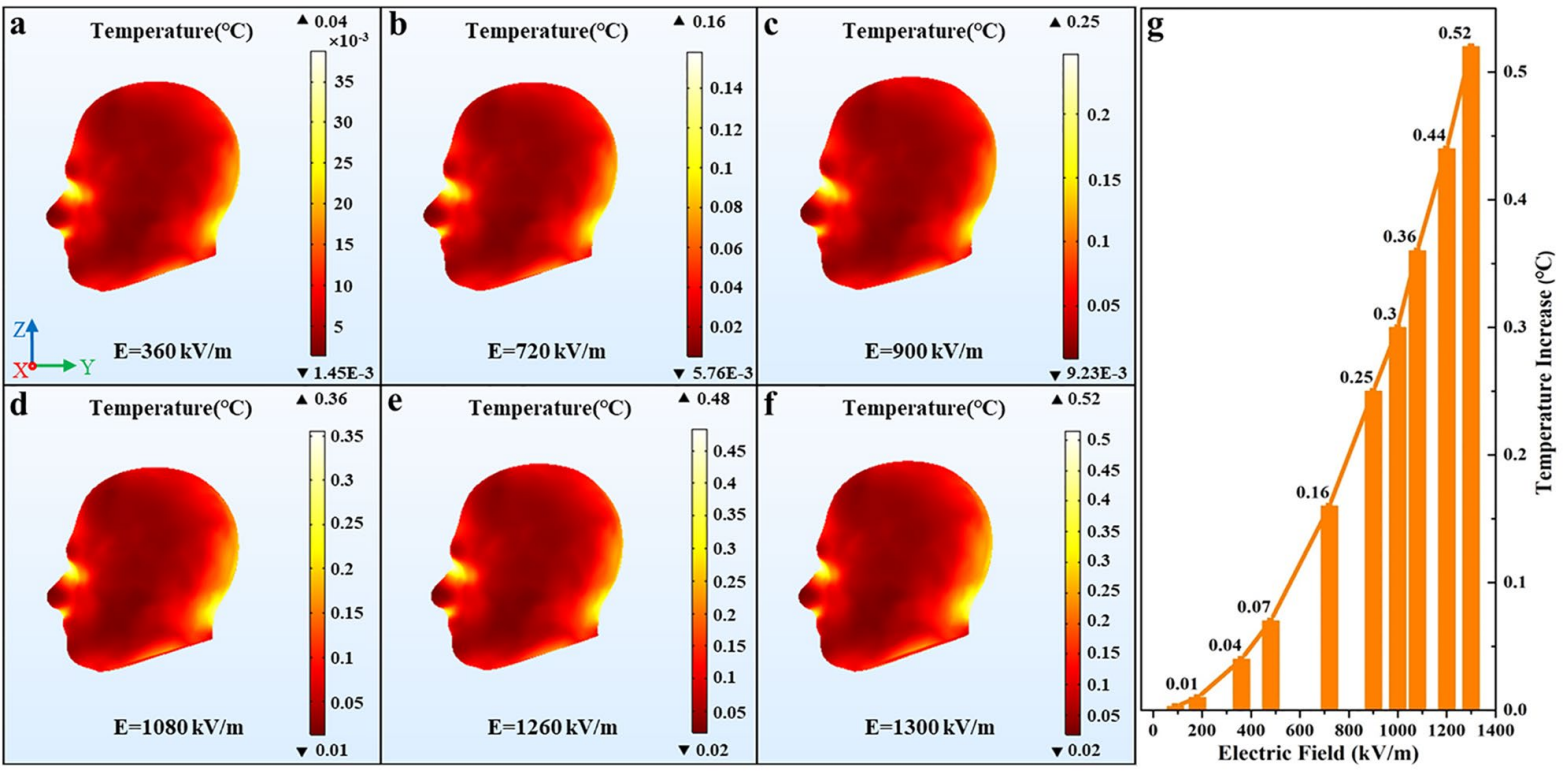
The temperature distribution in the human head and the variation curve of the temperature increase with different pulse field intensity. (**a**) E = 360 kV/m, (**b**) E = 720 kV/m, (**c**) E = 900 kV/m, (**d**) E = 1080 kV/m, (**e**) E = 1260 kV/m, (**f**) E = 1300 kV/m, (**g**) the variation curve of the temperature increase. We used COMSOL Multiphysics software, version 5.4 (https://www.comsol.com/comsol-multiphysics) for simulation results (Images **a**–**f**) and Origin software, version 8.0 (https://www.originlab.com/index.aspx?go=Products/Origin) for data analysis (Image **g**).

Temperature increases in the human head are obtained for different field intensity of EMPs. Figure 4 depicts the increases in temperature with the varied pulse field intensity, where, the pulse total number (n = 800), the pulse width (τ = 40 ns), and the pulse repetition frequency (f = 10 Hz) remained unchanged. Figure 4a–f show the head temperature distribution where each figure respectively indicates different pulse field intensity and Fig. 4b is the control group, which represents the SAM simulation with the pulse benchmark parameters. Figure 4g displays the variation curve of the temperature increase in the human head with the pulse field intensity. As depicted in Fig. 4g, the horizontal axis of the graph represents the pulse field intensity, and the vertical axis represents the highest temperature increase value in the head exposed to EMPs.

It can be seen from Fig. 4a–f that the temperature distribution in the human head almost coincide with that of the control group when the pulse field intensity is varied, that is, the temperature distribution in the human head when exposed to EMPs exerts slightly dependence on the pulse electric field. Figure 4g shows that the highest temperature rise value in the human head increases with the pulse field intensity when other parameters of EMP are constant, and it will exceed 0.5 °C when the pulse field intensity is stronger than 1300 kV/m, which may have a potential hazard to the human head.

### The dose–effect between the temperature increase of the human head and the pulse total number

**Figure 5:**
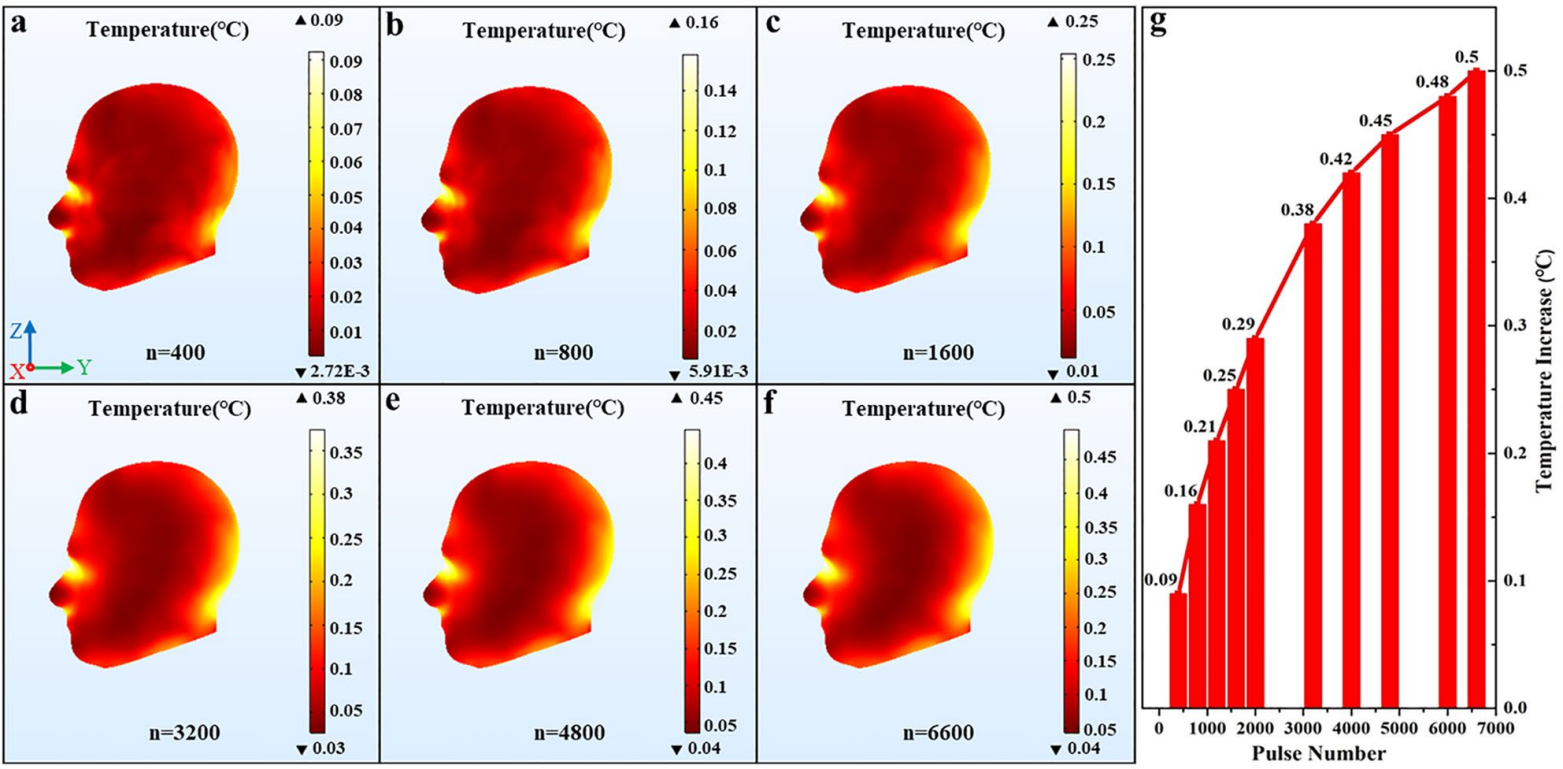
The temperature distribution in the human head and the variation curve of the temperature increase with different pulse total number. (**a**) n = 400, (**b**) n = 800, (**c**) n = 1600, (**d**) n = 3200, (**e**) n = 4800, (**f**) n = 6600, (**g**) the variation curve of the temperature increase. We used COMSOL Multiphysics software, version 5.4 (https://www.comsol.com/comsol-multiphysics) for simulation results (Images **a**–**f**) and Origin software, version 8.0 (https://www.originlab.com/index.aspx?go=Products/Origin) for data analysis (Image **g**).

Temperature increases in the human head are obtained for different pulse total number of EMPs. Figure 5 shows the increases in temperature with the varied pulse total number, where, the pulse field intensity (E = 720 kV/m), the pulse width (τ = 40 ns), and the pulse repetition frequency (f = 10 Hz) remained unchanged. Figure 5a–f show the head temperature distribution where each figure respectively indicates different pulse total number and Fig. 5b is the control group, which represents the SAM simulation with the pulse benchmark parameters. Figure 5g displays the variation curve of the temperature increase in the human head with the pulse total number. As shown in Fig. 5g, the horizontal axis of the graph represents the pulse total number, and the vertical axis represents the highest temperature increase value in the head exposed to EMPs.

It can be seen from Fig. 5a–f that the temperature distribution in the human head almost coincide with that of the control group when the pulse total number is varied, that is, variations of the pulse total number have no effects on the temperature distribution in the human head when exposed to EMPs. Figure 5g shows that the temperature rise in the human head increases with the pulse total number when other parameters of EMP are constant, and the highest temperature rise value in the human head changes significantly when the pulse total number is smaller than 4800, otherwise, it remains slight changes, and it will exceed 0.5 °C when the pulse total number is larger than 6600, thus an adverse effect on the human body may occur.

### The dose–effect between the temperature increase of the human head and the pulse width

**Figure 6:**
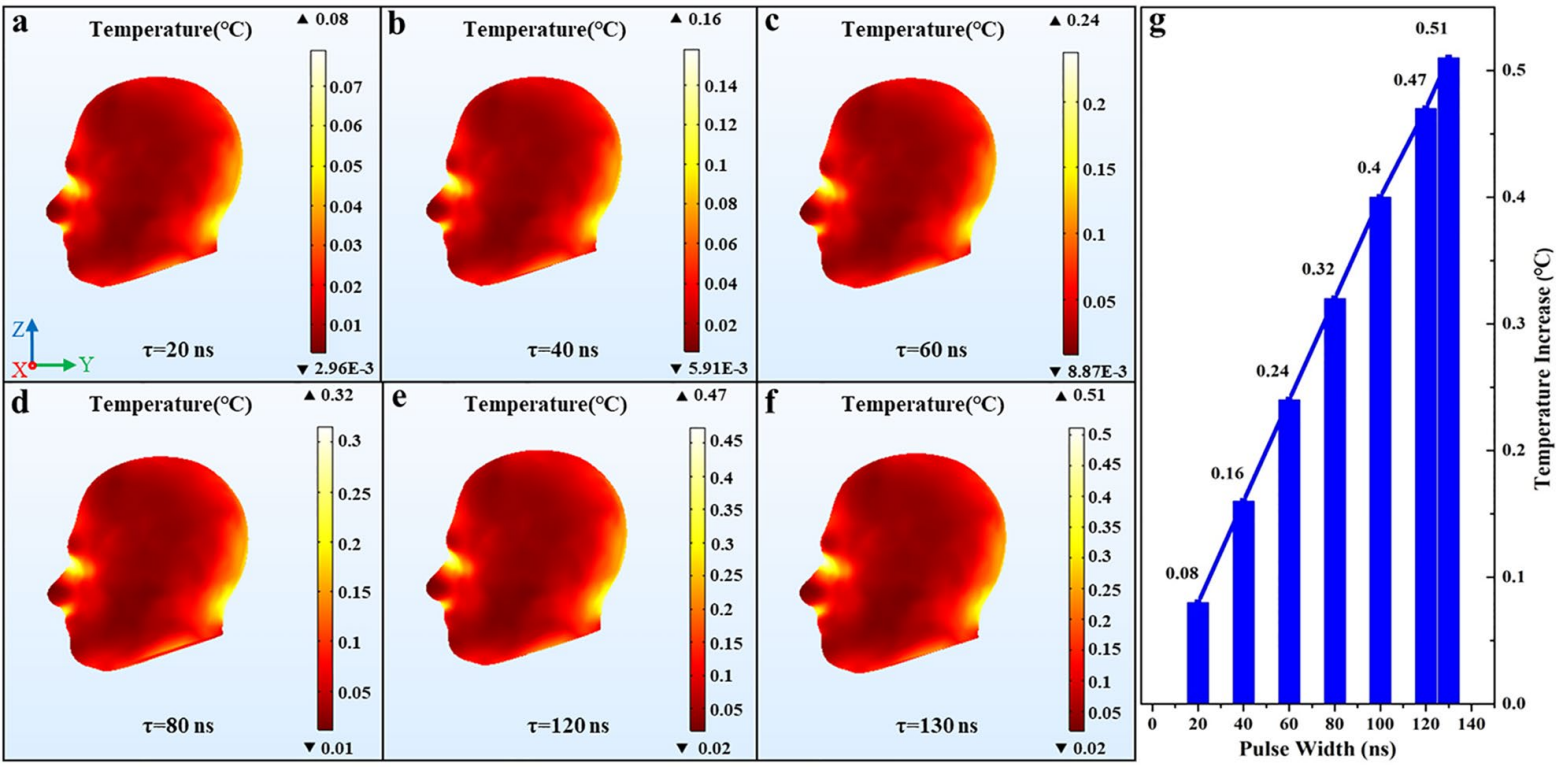
The temperature distribution in the human head and the variation curve of the temperature increase with different pulse width. (**a**) τ = 20 ns, (**b**) τ = 40 ns, (**c**) τ = 60 ns, (**d**) τ = 80 ns, (**e**) τ = 120 ns, (**f**) τ = 130 ns, (**g**) the variation curve of the temperature increase. We used COMSOL Multiphysics software, version 5.4 (https://www.comsol.com/comsol-multiphysics) for simulation results (Images **a**–**f**) and Origin software, version 8.0 (https://www.originlab.com/index.aspx?go=Products/Origin) for data analysis (Image **g**).

Temperature increases in the human head are obtained for different pulse width of EMPs. Figure 6 demonstrates the increases in temperature with the varied pulse width, where, the pulse field intensity (E = 720 kV/m), the pulse total number (n = 800), the pulse repetition frequency (f = 10 Hz) remained unchanged. Figure 6a–f show the head temperature distribution where each figure respectively indicates different pulse width and Fig. 6b is the control group, which represents the SAM simulation with the pulse benchmark parameters. Figure 6g displays the variation curve of the temperature increase in the human head with the pulse width. As shown in Fig. 6g, the horizontal axis of the graph represents the pulse width, and the vertical axis represents the highest temperature increase value in the head exposed to EMPs.

It can be seen from Fig. 6a–f that the temperature distribution in the human head almost coincide with that of the control group when the pulse width is varied, that is, the temperature distribution in the human head when exposed to EMPs exerts slightly dependence on the pulse width. Figure 6g shows that the temperature rise in the human head increases with the pulse width when other parameters of EMP are constant, and it will exceed 0.5 °C when the pulse width is greater than 130 ns, which may cause adverse effects on the human health.

## Discussion

In this paper, the temperature increases in the human head when exposed to EMPs have been investigated based on commercial software called COMSOL Multiphysics. The simulation results show that the highest temperature rise value in the human head is 0.16℃ with the pulse benchmark parameters (E = 720 kV/m, n = 800, τ = 40 ns, f = 10 Hz), and the temperature increases in the human head are non-thermal effects within the studied EMP parameters. The temperature rise values are large close to the head surface facing the incident wave, and drop rapidly inside the head. The temperature distribution profiles with different pulse parameters have almost similar characteristics and the highest temperature rise value in the human head increases with the pulse field intensity, pulse total number, and pulse width. Besides, EMPs exposure may exert adverse effects on the human head when the pulse field intensity is stronger than 1300 kV/m, the pulse total number is larger than 6600 pulses, and the pulse width is greater than 130 ns, which were defined as the safety threshold of head exposure to EMPs. Additionally, this paper would offer datasets to characterize the relationship between the human head temperature and EMP parameters that can inform further studies that utilize high-fidelity models and this study would be of great theoretical significance for further researches on the optimal EMP parameters to open the BBB to the greatest extent within a safe range.

## Supplementary Information

Supplementary Video 1.

Supplementary Figure S1.

## Data Availability

The datasets generated or analysed during the current study are available from the corresponding author on reasonable request.

## References

[1] Altar CA, Vawter MP, Ginsberg SD (2009). Target identification for CNS diseases by transcriptional profiling. Neuropsychopharmacology.

[2] Genc S, Zadeoglulari Z, Fuss SH, Genc K (2012). The adverse effects of air pollution on the nervous system. J. Toxicol..

[3] Oberdörster G, Utell MJ (2002). Ultrafine particles in the urban air: To the respiratory tract—and beyond?. Environ. Health Perspect..

[4] Arora RS (2010). Are reported increases in incidence of primary CNS tumours real? An analysis of longitudinal trends in England, 1979–2003. Eur. J. Cancer.

[5] Caldarella A, Crocetti E, Paci E (2011). Is the incidence of brain tumors really increasing? A population-based analysis from a cancer registry. J. Neuro-Oncol..

[6] Deorah S, Lynch CF, Sibenaller ZA, Ryken TC (2006). Trends in brain cancer incidence and survival in the United States: Surveillance, epidemiology, and end results program, 1973 to 2001. Neurosurg. Focus..

[7] McKean-Cowdin R (2013). Trends in childhood brain tumor incidence, 1973–2009. J. Neuro-Oncol..

[8] Nomura E, Ioka A, Tsukuma H (2011). Trends in the incidence of primary intracranial tumors in Osaka, Japan. Jpn. J. Clin. Oncol..

[9] Patel S (2014). Are pediatric brain tumors on the rise in the USA? Significant incidence and survival findings from the SEER detabase analysis. Child’s Nerv. Syst..

[10] Bellavance MA, Blanchette M, Fortin D (2008). Recent advances in blood-brain barrier disruption as a CNS delivery strategy. AAPS J..

[11] Pardridge WM (2007). Blood–brain barrier delivery of protein and non-viral gene therapeutics with molecular Trojan horses. J Control Release.

[12] Pathan SA (2009). CNS drug delivery systems: Novel approaches. Recent Pat. Drug Deliv. Formul..

[13] Abbott NJ (2013). Blood–brain barrier structure and function and the challenges for CNS drug delivery. J. Inherit. Metab. Dis..

[14] Ding GR (2009). Effect of electromagnetic pulse exposure on brain micro vascular permeability in rats. Biomed. Environ. Sci..

[15] Wang Q (2003). The study of dose-response relationship of pulsed electromagnetic radiation on rat blood-brain-barrier. Chin. J. Dis. Control Prev..

[16] Gao P (2017). Effects of Electromagnetic Radiation with Different Parameters on Permeability of Blood Brain Barrier.

[17] Vecchia P (2009). Exposure to High Frequency Electromagnetic Fields, Biological Effects and Health Consequences (100 kHz–300 GHz).

[18] Challis LJ (2005). Mechanisms for interaction between RF fields and biological tissue. Bioelectromagn. Suppl..

[19] Giuliani L, Soffritti M (2010). Non-thermal effects and mechanisms of interaction between electromagnetic fields and living matter. Eur J Oncol..

[20] COMSOL Multiphysics. Specific Absorption Rate (SAR) in the Human Brain. https://www.comsol.com/model/specific-absorption-rate-sar-in-the-human-brain-2190 (2018).

[21] Wessapan T, Srisawatdhisukul S, Rattanadecho P (2011). Numerical analysis of specific absorption rate and heat transfer in the human body exposed to leakage electromagnetic field at 915 MHz and 2450 MHz. ASME J. Heat Transf..

[22] Spiegel RJ (1984). A review of numerical models for predicting the energy deposition and resultant thermal response of humans exposed to electromagnetic fields. IEEE Trans. Microw. Theory Tech..

[23] Shen W, Zhang J (2005). Modeling and numerical simulation of bioheat transfer and biomechanics in soft tissue. Math. Comput. Model..

