# Supplementary material for: Effects of pulse parameters on the temperature distribution of a human head exposed to the electromagnetic pulse
Source: Sci Rep. 2021 Nov 25;11:22938. doi: 10.1038/s41598-021-02396-8 (PMC8617282; doi:10.1038/s41598-021-02396-8)
Supplement: Supplementary file 2 — Supplementary Figure S1. [file 41598_2021_2396_MOESM2_ESM.pdf]

Supplementary Information

Effects of pulse parameters on the temperature distribution of a human head exposed to the electromagnetic pulse

Shan Wang[1], Zhongguo Song[1,*], Yanning Yuan[1], Guozhen Guo[2], Jianjun Kang[3]

[1] Faculty of Automation and Information Engineering, Xi'an University of Technology, Xi'an, 710048, China

[2] Department of Radiation Biology, Air Force Medical University, Xi'an, 710032, China

[3] Xi'an Jiushuo Institute of Biotechnology, Xi'an, 710065, China

* songzhongguo@xaut.edu.cn

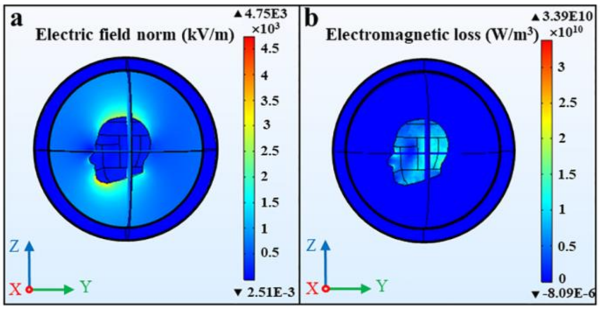

Figure S1. The multi-section electric field in the brain exposed to an EMP at 250 MHz and the electromagnetic loss. (a) The electric field norm, (b) The electromagnetic loss.

Supplementary Video. The multi-section background electric field.
